# Exploring Modality Switching Effects in Negated Sentences: Further Evidence for Grounded Representations

**DOI:** 10.3389/fpsyg.2013.00093

**Published:** 2013-02-28

**Authors:** Lea A. Hald, Ian Hocking, David Vernon, Julie-Ann Marshall, Alan Garnham

**Affiliations:** ^1^Donders Institute for Brain, Cognition and Behaviour, Radboud University NijmegenNijmegen, Netherlands; ^2^Applied Social Sciences, Canterbury Christ Church UniversityCanterbury, Kent, UK; ^3^Cambridge CognitionCambridge, UK; ^4^School of Psychology, University of SussexFalmer, East Sussex, UK

**Keywords:** ERP, N400, negation, embodiment, language processing, veracity, modality, modality switch effect

## Abstract

Theories of embodied cognition (e.g., Perceptual Symbol Systems Theory; Barsalou, 1999, 2009) suggest that modality specific simulations underlie the representation of concepts. Supporting evidence comes from modality switch costs: participants are slower to verify a property in one modality (e.g., auditory, BLENDER-loud) after verifying a property in a different modality (e.g., gustatory, CRANBERRIES-tart) compared to the same modality (e.g., LEAVES-rustling, Pecher et al., 2003). Similarly, modality switching costs lead to a modulation of the N400 effect in event-related potentials (ERPs; Collins et al., 2011; Hald et al., 2011). This effect of modality switching has also been shown to interact with the veracity of the sentence (Hald et al., 2011). The current ERP study further explores the role of modality match/mismatch on the processing of veracity as well as negation (sentences containing “not”). Our results indicate a modulation in the ERP based on modality and veracity, plus an interaction. The evidence supports the idea that modality specific simulations occur during language processing, and furthermore suggest that these simulations alter the processing of negation.

## Introduction

When reading, it has been demonstrated that switching from a sentence primarily describing information in one modality to text describing information in another modality leads to an increase in processing cost (the modality switch effect, Pecher et al., 2003). Similar modality switching effects have been found across both conceptual and perceptual processing tasks (e.g., Spence et al., 2001; Marques, 2006; Vermeulen et al., 2007; Van Dantzig et al., 2008). For instance, Pecher et al. (2003) presented participants with short sentences one after another that consisted of a concept followed by a modal property (they used audition, vision, taste, smell, touch, and action). Unknown to the participants, the sentences were actually in pairs that either matched or mismatched in modality. For example, a matched auditory modality would be *Leaves can be rustling* followed by *A blender can be loud* vs. mismatched gustatory-auditory modalities *Cranberries can be tart* followed by *A blender can be loud*. Although participants were unaware that the sentences were paired, reaction times to verify whether the final word was a typical property of the concept (e.g., that loud was a typical property of the concept blender, property verification task) were faster and more accurate when the pairs of sentences matched in modality compared to pairs that mismatched. Recent evidence indicates that the modality switch effect also results in a modulation of event-related potentials (ERPs), specifically a modulation of the N400 effect (e.g., Collins et al., 2011; Hald et al., 2011; described in more detail below). An N400 is a negative deflection in the ERP that begins around 250 ms post stimulus onset and peaks around 400 ms. It is typically larger across the centro-parietal electrode sites. Broadly speaking, an N400 effect has been shown to occur to any meaningful stimuli, such as a word, picture, or sign in sign language, that is either less expected or anomalous based on the particular context or knowledge a person has about the situation (see Kutas and Federmeier, 2011, for a recent review). Typically, the modality switch effect has been explained by the idea that our conceptual system is grounded in modality specific or embodied simulations (e.g., Barsalou, 1999; Glenberg and Robertson, 1999, 2000; Zwaan, 2004; Zwaan and Madden, 2005; but, see also Louwerse and Connell, 2011, for a discussion of the influence of statistical regularities on this effect). That is, the meanings of linguistic stimuli rely on modality specific sensorimotor information or simulations. Within this framework it has been proposed that the switching cost is due to changing from one modality specific brain system to another.

The goal of the current study is to explore the modulation of the modality switch N400 effect. Specifically, we aim to explore whether this effect is sensitive to linguistic and semantic markers. By adding specific linguistic and semantic properties to the typical modality switch paradigm, we hope to better understand the timing and the automaticity of embodied cognition effects during language processing. An understanding of the timing and automaticity of embodied effects on language comprehension is necessary for building a better model of the role of embodied cognition in language processing. To realize this goal, we have added the factors negation and veracity to a typical modality switch paradigm. Additionally, we have implemented a different task for the participants.

Typically, studies looking at the modality switch effect have utilized the property verification task. As discussed above, participants have to verify that a property is “usually true” or “usually false” of a particular concept (e.g., Pecher et al., 2003). In order to explore the role of veracity and negation within this paradigm, we decided to implement the sentence verification task. Sentence verification is similar to property verification. In sentence verification, sentences are presented and subjects respond with a true or false judgment at the end of the sentence. Comparing items that work in both tasks it is clear that some items can be almost identical (“A blender can be loud”), while others can only be used in the sentence verification (“A baby drinks milk”). The advantage of using sentence verification rather than property verification is that the former has a long history of being used to investigate veracity and negation both behaviorally (for a review of the sentence verification task, see Carpenter and Just, 1975) and in ERP experiments (e.g., Fischler et al., 1983).

### Why veracity and negation?

Veracity and negation have been studied outside of the domain of embodied cognition extensively. For veracity, it has been consistently shown that when participants are asked to judge the veracity of a sentence, true sentences are verified faster than false sentences (for example, Trabasso et al., 1971; Clark and Chase, 1972; Wason, 1980). The primary explanation for this is that readers match the relevant conceptual information provided in the sentence to either the external situation (when the task requires comparing the veracity of a sentence to a given picture) or their general world knowledge (when the task involves sentences only). When the conceptual information and external situation/world knowledge are incongruent (a false sentence) there is a slowing of responses (Carpenter and Just, 1975; see also Fischler et al., 1983). Similarly, a corresponding modulation of the N400 effect using ERPs has been seen for false sentences (e.g., Fischler et al., 1983; Hagoort et al., 2004). However, whether this comparison between information in the sentence and general world knowledge relies on an embodied representation of the sentence in order to judge veracity is not clear. Furthermore, to our knowledge no model of embodied cognition has adequately described how this comparison process may happen. This is a point we return to.

Across many experiments it has been found that sentences containing negation are verified or read slower than sentences that do not contain negation (Wason, 1959, 1980; Trabasso et al., 1971; Clark and Chase, 1972; Carpenter and Just, 1975; Singer, 2006). Furthermore, an interaction of negation and veracity has been replicated many times. Essentially, true affirmative sentences (*Six is an even number*) are verified or read faster than false affirmative sentences (*Six is an odd number*), while true negative sentences (*Six is not an odd number*) are verified or read slower than false negative sentences (*Six is not an even number*). “Two-step” theories of negation suggest that the reason that determining the truth value of a negated sentence is particularly difficult is because people have to first suppose an “inner proposition” (*Six is an odd number*) before they can apply the negation term to compute the truth value (e.g., Kintsch, 1974; Carpenter and Just, 1975; Clark and Clark, 1977; see Kaup et al., 2007a for review). A related finding has also been shown using ERPs. Specifically, negative sentences lead to a different pattern in the N400 compared to affirmative sentences (Fischler et al., 1983). Although the typical finding with affirmative sentences is a larger N400 for false, semantically incorrect sentences, for sentences containing negation it is the correct, semantically coherent sentences that lead to a larger N400 amplitude. It is often assumed that this N400 reflects the “inner proposition,” prior to the point negation is actually integrated (e.g., Fischler et al., 1983). In sum, the results with both ERPs and reading times suggest that true negated sentences are more difficult to process than false negated sentences.

The only exception to the processing difficulties and ERP pattern for negation appears to be when a context is used that supports the use of negation (e.g., Wason, 1965; Wales and Grieve, 1969; Glenberg et al., 1999; Garton and Robertson, 2003; Nieuwland and Kuperberg, 2008; Tian et al., 2010). When there is an appropriate context, the processing of negation appears to be processed in a manner similar to affirmative sentences. That is, the pattern of reaction times and ERPs look no different from what you would expect with an affirmative sentence.

Interestingly, both false sentences and negated sentences have presented complications in terms of how they are represented in an embodied framework. Barsalou (1999) describes negation as being closely related to the concept of truth. Although both negation and falsity are discussed in the context of comparing a sentence to a situation (or picture) as opposed to background knowledge about the topic, essentially Barsalou proposes that both are represented by creating absent mappings within a simulation between the relevant entities. Specifically, when making a simulation of the information in a sentence, either a false sentence or a negated sentence can lead to a simulation that fails. The marking of that failure, noting the absence of a binding between the relevant entities is what underlies the representation. For example, when simulating the sentences “It’s false that there is a balloon above the cloud” and “It’s true that there is not a balloon above the cloud” noting the absence of a binding between balloon and cloud is necessary in the simulation of both sentences. Based on this explanation, one might expect to find a similar ERP modulation relative to modality switching for both false sentences and sentences containing negation since according to this embodied cognition framework, they are simulated/represented in the same manner. However, the possible mechanisms of embodied veracity and negation processing have not been well explored. It is still an open question whether, and especially how, an embodied representation could support veracity judgment and negation processing.

Finding that modality switching interacts with veracity and/or negation would help us better understand how sentence processing relies on embodied cognition. Furthermore, it is possible that we see differential ERP modulation for modality switching in sentences containing negation compared to false sentences. Finding such an effect would indicate that the Barsalou (1999) account of negation and false sentences is insufficient. For these reasons, we have implemented the sentence verification task to explore modality switching in true and false sentences that contain negation.

Following a brief review of the small amount of research that exists on the embodied nature of veracity and negation, details of the current experiment will be discussed.

### Veracity and modality switching with affirmative sentences

The study most relevant to the current study is a recent one by Hald et al. (2011). The authors explored veracity and the modality switch effect with *affirmative* sentences. In this study, the experimental materials included both true and false modality matched and mismatched pairs (see Table 1).

**Table 1:** **Example tactile materials from Hald et al. (2011)**.

Veracity	Modality switch	Modality context	Target sentence
True	Mismatched	A leopard is spotted	A peach is **soft**
	Matched	An iron is hot	A peach is **soft**
False	Mismatched	A leopard is spotted	A peach is **hard**
	Matched	An iron is hot	A peach is **hard**

*Critical words are shown here in bold for clarification*.

For example, the ERPs were compared for *soft* (vs. *soft*) and hard (vs. *hard*) depending on the modality match/mismatch. Additionally, the ERPs to true vs. false sentences (*soft* vs. *hard*) were compared within match and within mismatch conditions.

As discussed above, in traditional ERP studies a consistently larger amplitude N400 is typically seen for words that complete a sentence in such a way as to make the truth value of the sentence false (for example, at the final word when comparing *a ham is blue* vs. *a ham is pink*; Fischler et al., 1983). However, it is unclear how or whether a match or mismatch in modality may affect the processing related to veracity in such cases. It has been suggested that when a false sentence is read, simulation fails. That is, the meaning of the sentence cannot be successfully mapped onto reality (Barsalou, 1999). Presumably at this point a new simulation is performed, somehow grounded in the failed simulation (see Barsalou, 1999 for more details on this argument). However, whether and how this actually occurs is unclear. One of the purposes of looking at false sentences in the modality switch paradigm was to shed light on the process of understanding a false sentence, and to explore how this may occur according to embodied models of cognition.

The results of Hald et al. (2011) indicated a different pattern of results for true and false sentences. Specifically, for the true sentences, switching modalities elicited a greater negativity across anterior electrodes as early as 160 ms after the onset of the critical word (*soft*). This effect was seen in three time windows: from 160 to 215 ms, from 270 to 370 ms, and again from 500 to 700 ms (see also Collins et al., 2011 for similar ERP results using the property verification task). However, for the false sentences, no significant effect of modality switching was seen. When comparing the effect of veracity (*soft* vs. *hard*) within the mismatch condition (A leopard is spotted – A peach is *soft/hard*), a typical N400 was seen for false sentences compared to true sentences. However, when the modality matched (An iron is hot – A peach is *soft/hard*), no effect of veracity was found. In so far as the N400 amplitude reflects difficulty in processing, this result suggests that the construction of a simulation in one modality aided the matching modality simulation of the target sentence. Possibly this led to the false sentences being no more difficult to comprehend than the true sentences.

This study suggests that veracity judgments are grounded in an embodied manner. That is, when a saving can be made in the embodied simulation of the sentence by having the same modality simulated twice in a row, this leads to improved ability to judge the veracity of false sentences. Although this result indicates that embodied cognition is important for the processing of semantics related to judging truth value, it does not address whether embodied cognition plays a role in more linguistically marked aspects of language, namely negation.

### Negation, embodied cognition, and context

Evidence supporting the idea that at least at a late point in time, negation processing relies on embodied simulations comes from Kaup et al. (2007b) to Kaup and Zwaan (2003). In both studies, they assessed the accessibility of a word that was either negated or not using a recognition task. For example, in Kaup and Zwaan (2003) participants were presented with short discourses that contained a color term that was either mentioned within the scope of a negative context or not, which then led to a situation where the color was either present or not. Participants had to determine whether a color term was in the previous sentence (probe recognition task). For example, for the sentence *Sam was relieved that Laura was not wearing her pink dress*, the probe word *pink* was presented after the sentence at an early and late time delay. In this example, the color term pink was within the scope of negation and the color pink would not actually be present in the situation described. Results indicated that at the early delay probe point (500 ms delay after sentence end) response times were slower when the color term had been negated. In the late time delay (1500 ms) the response time to the color term was influenced by the content of the situation (whether the situation described meant the color would be part of the situation, for example *Sam wished that Laura was not wearing her pink dress*, where the pink dress is part of the situation vs. *Sam was relieved that Laura was not wearing her pink dress*, where the pink dress is not part of the situation). This experiment, as well as others (e.g., Kaup et al., 2006) support the general idea that a simulation is made that notes the absence of negated information, making that information more difficult to retrieve. However, these studies only speak to the eventual representation of the negation, rather than to the ongoing process of comprehending/representing the negation as the sentence unfolds. Furthermore, these studies support the idea that something like a simulation is built, but do not address the specifics of how/whether the simulation is grounded in perceptual, action, and emotional information (although, see Kaup et al., 2006, for results suggesting that spatial information is part of this embodied simulation, at least at a delayed point). The current study will specifically address the role of perceptual modalities in the online processing of negation. However, as discussed earlier, when it comes to negation, context matters.

Whether an early effect of negation processing appears is largely related to the context in which negation is used, that is when the context supports the use of negation (see Glenberg et al., 1999). The goal of the current study was to better understand what the role of a modality match/mismatch may be on the ongoing processing of negation. The current study was designed to answer the following questions: first, can we find a modality switch effect with sentences containing negation? If we do see such an effect, this suggests that sentences containing negation are grounded in perceptual systems^1^. Secondly, since context has been shown to affect the processing of negation, can modality information similarly change the processing of negation? Given that Hald et al., 2011 found that modality matching aided the processing of false sentences, could modality matching similarly facilitate negation processing?

### The current study

The current study is based on the Hald et al. (2011) study in Section “Veracity and Modality Switching with Affirmative Sentences.” However, in addition to exploring the effect of veracity and modality switching, here the target sentences all included negation (see Table 2 in Materials and Methods for example stimuli).

**Table 2:** **Example materials for tactile and visual modality**.

Veracity	Modality switch	Modality context	Target sentence
**Tactile target sentence example**			
True	Mismatched	A kingfisher is bright blue	A marble isn’t **soft**
	Matched	A summer night is balmy	A marble isn’t **soft**
False	Mismatched	A kingfisher is bright blue	A marble isn’t **hard**
	Matched	A summer night is balmy	A marble isn’t **hard**
**Visual target sentence example**			
True	Mismatched	A light bulb is very hot	Rice isn’t **black**
	Matched	A giraffe is spotted	Rice isn’t **black**
False	Mismatched	A light bulb is very hot	Rice isn’t **white**
	Matched	A giraffe is spotted	Rice isn’t **white**

*Critical words are shown here in bold for clarification*.

For the modality switch effect in negated sentences, we compared the ERPs time locked to the identical word in a sentence, depending on whether the previous context sentence matched or mismatched in modality. For example, we compared the ERP to the word *soft* in the true sentence *The marble isn’t soft* when it was preceded by a modality matched sentence (*A summer night is balmy*) vs. when it was preceded by a modality-mismatched sentence (*A kingfisher is bright blue*; see Materials and Methods for details about the sentence materials). Finally, to explore the effect of modality and negation on veracity we compared the ERPs to true vs. false sentences within the match condition and then within the mismatch condition (for example comparing *The marble isn’t soft* vs. *The marble isn’t hard* when the previous sentence matched in modality.

According to embodied accounts of cognition/language processing (e.g., Barsalou, 1999, 2009; Zwaan and Madden, 2005), as well as the previous results discussed above, we expect to see an effect of modality switching in the true sentences. However, the negation may cause this effect to be delayed. This would be in line with the delayed embodied effects in negated sentences found by Kaup et al. (2007b). For the false sentences, it is unclear whether an effect of modality switching will be seen at all given the previous results (e.g., Hald et al., 2011). Finally, it may be the case that modality matching might actually aid the processing of negation, as has been seen with discourse context (e.g., Nieuwland and Kuperberg, 2008). If that is the case, then we would expect negated false sentences to elicit greater N400 amplitudes than negated true sentences when preceded by a modality matched sentence. For the mismatched condition we would expect the true negated sentences to elicit a larger N400 amplitude than the false negated sentences, since this pattern of results is typically found when negated sentences are presented out of context (in line with Fischler et al., 1983). Overall, by examining the modality switch effect in combination with veracity and negation, a richer understanding of the parameters by which embodied cognition influences language comprehension should be achievable.

## Materials and Methods

### Participants

Sixteen participants were initially recruited from the Psychology undergraduate cohort attending Canterbury Christ Church University and took part in the study. Of these three were eliminated during the filtering of target EEG events due to a large amount of data loss (i.e., a loss of more than one third of target events). A further two participants were excluded from the final analysis because their EEG recordings exhibited excessive artifacts resulting in the loss of a large number of trials (i.e., a loss of more than one third of trials), resulting in a final sample of eleven participants (seven females; aged 18–32, mean 21.1; four males; aged 18–26, mean 22.5). Participants were awarded course credit for completing the study and all had normal or corrected to normal vision, were right handed, native English speakers, and had not been diagnosed with reading or speaking difficulties.

Ethical approval for the study was granted by Canterbury Christ Church University’s Faculty Research Ethics Committee and all participants provided written consent prior to taking part in the study.

### Stimulus material and design

Materials comprised 160 pairs of experimental sentences consisting of an initial sentence, referred to as the Modality Context sentence and a second Target sentence. The Modality Context sentences were always true non-negated statements and were evenly divided into those that described either a visual (50%) or haptic property (50%) of an object. The Modality Context sentences were a subset of items that have been previously rated as more salient in one modality than others (see Pecher et al., 2003; Van Dantzig et al., 2008; Lynott and Connell, 2009).

The target sentences were always negated (e.g., Rice isn’t black/white) and in half of the trials their modality matched that of the Modality Context sentence, with veracity of the target sentence equally balanced. Hence, modality-match/-mismatch and target sentence veracity were fully crossed creating 40 pairs of modality matched true sentences, 40 pairs of modality matched false sentences, 40 pairs of modality-mismatched true sentences, and 40 pairs of modality-mismatched false sentences.

False versions of the negated target sentences were created using words that were independently rated as the opposite of the salient modality feature of the object (see Hald et al., 2011). For example, in the true negated visual target sentence “Rice isn’t black” the salient visual feature of “black” was replaced with “white” (see Table 2 for example stimuli; a full list of negated sentences is available on request).

To ensure that there was an equal number of affirmative and negative sentences an additional 160 filler sentences were constructed. These comprised 80 affirmative and 80 negative sentences. Half of the filler sentences contained strong modality related properties, using tactile, visual, auditory, and gustatory modality related information. The remaining half was not based on modality specific information but merely contained highly related words conveying false information (e.g., “A ball is refereed”; see Pecher et al., 2003 for similar use of semantically related items). However, it was not possible to match the number of sentence pairs that were context negative-target affirmative with those that were context affirmative-target negative. Such a procedure would have required an additional extra 80 sentence pairs which would also have increased the duration of the task and in all likelihood led to a reduction in participant motivation and engagement levels. Thus, given that the participant remained unaware of the fact that sentences were presented in pairs it seemed more important to control for the absolute number of affirmative and negative sentences and true and false sentences.

The critical words were matched on a number of measures including: (i) word log (lemma) frequency (true-matched modality: 2.37; true-mismatched modality: 2.37; false-matched modality: 2.32; and false-mismatched modality: 2.32, from Baayen et al., 1993); (ii) word length (true-matched modality: 4.5 letters; true-mismatched modality: 4.5 letters; false-matched modality: 4.7 letters; and false-mismatched modality: 4.7 letters); and (iii) word class (all adjectives). In addition, none of the critical words was over 12 letters in length.

The pairs of sentences were presented in a pseudo-randomized order specific to each participant (created using Mix; Van Casteren and Davis, 2006) using a fully within participants design. The use of a within participants design meant that the findings from this study could be easily compared to previous similar designs (e.g., Fischler et al., 1983; Pecher et al., 2003; Hald et al., 2011).

### Procedure for the ERP study

After reading an information sheet, participants completed a short questionnaire asking about language background, basic health, and handedness. They then completed a standard consent form and began the experiment. Each participant was tested individually in a quiet room, seated in a comfortable chair approximately 70 cm from the computer monitor and were asked not to move or blink during the presentation of the sentences. Participants were asked to read each sentence for comprehension and decide whether it was true or false.

The stimuli were presented using the E-Prime 2.0 (Schneider et al., 2002) stimuli presentation platform. Each session began with a practice block of 10 sentences, which were similar in nature to the experimental items. At the end of the practice block, the participant had the opportunity to ask questions relating to the task. The remaining sentences were then split into six blocks, each lasting for approximately 12 min, with a short break between blocks. Each block began with two filler items, which were similar in nature to the experimental items. These filler items were included to minimize the potential loss of data due to artifacts resulting from beginning a task.

Each trial began with a fixation point (“+++”) displayed for 1 s in the center of the screen. Participants were told that they could blink their eyes during the fixation display if needed, but to be prepared not to blink during the upcoming sentence. After a variable time delay (randomly varying across trials from 300 to 450 ms), the sentence was presented word by word in white lowercase letters (Courier New, 18-point font) against a black background. The first word and any proper noun were capitalized and the final word of each sentence was followed by a full stop. Words were presented for 200 ms with a stimulus onset asynchrony of 500 ms. Following presentation of the final word in each sentence the screen remained blank for 1000 ms after which three question marks appeared, along with the text, “1:true” and “5:false.” Participants needed to respond by pressing either the “1” or the “5” on the number keypad of a standard keyboard to indicate whether they thought the sentence was true or false. The association between number and veracity was counterbalanced so that for all participants, half of the time the number 1 indicated true and half the time the number 5 indicated true. If participants responded incorrectly the feedback message “Wrong Answer” was displayed and if they took more than 3000 ms to respond the feedback message “Too Slow” was displayed. Exactly the same presentation procedure was used for context and target sentences so that participants remained unaware that sentences were presented in pairs. Following the experiment all participants were debriefed.

### EEG recording and analysis

The EEG was recorded using a 64-channel WaveGuard Cap utilizing sintered Ag/AgCl electrodes connected to an ANT amplifier (ANT, Enschede, Netherlands). An average reference was used. The electrodes were placed according to the 10–20 standard nomenclature (Jasper, 1958) over midline (FPz, Fz, FCz, Cz, CPz, Pz, POz, and Oz) lateral (Fp1, Fp2, AF3, AF4, AF7, AF8, F1, F2, F3, F4, F5, F6, F7, and F8), fronto-central (FC1, FC2, FC3, FC4, FC5, and FC6), central (C1, C2, C3, C4, C5, and C6), temporal (FT7, FT8, T7, T8, TP7, and TP8), centro-parietal (CP1, CP2, CP3, CP4, CP5, and CP6), parietal (P1, P2, P3, P4, P5, and P6), and occipital (PO3, PO4, PO5, PO6, PO7, PO8, O1, and O2) positions. The signals were digitized online with a sampling frequency of 512 Hz and bandpass filtered from 0.01 to 100 Hz. Electrode impedance was maintained below 10 kΩ.

Analysis was conducted using ASA (ANT, Enschede, Netherlands) software. EEG data were initially screened for potential artifacts in a critical window ranging from −100 to 1000 ms post stimulus onset. Trials containing artifacts were excluded from further analysis, which resulted in 90.83% of epochs being included.

## Results

**Figure 1:**
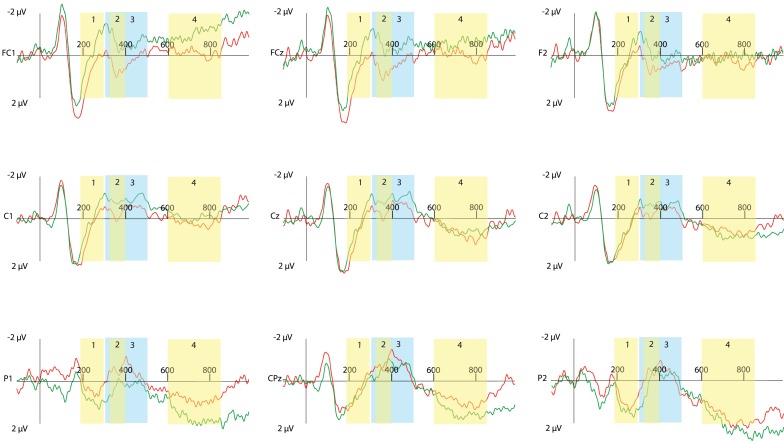
**Event-related potential traces for true sentences for nine selected sites across the scalp, time locked to onset of the critical word (presented at 0 ms)**. Negative activation is plotted up. The red lines show the True-Mismatched condition, the green line shows the True-Matched condition. The limits of each of the four time windows for analysis are indicated (1 = 190–300 ms; 2 = 325–400 ms; 3 = 300–500 ms; 4 = 600–850).

**Figure 2:**
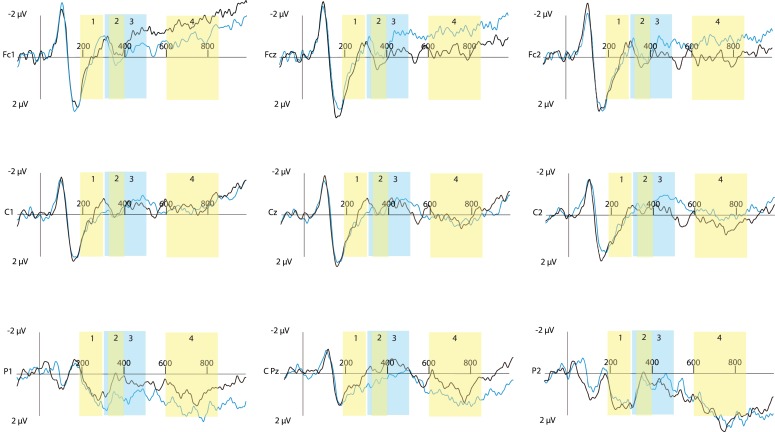
**Event-related potential traces for false sentences for nine selected sites across the scalp, time locked to onset of the critical word (presented at 0 ms)**. Negative activation is plotted up. The blue lines show the False-Mismatched condition, the black line shows the False-Matched condition. The limits of each of the four time windows for analysis are indicated (1 = 190–300 ms; 2 = 325–400 ms; 3 = 300–500 ms; 4 = 600–850).

An overview of nine representative electrodes (out of 64 total electrodes) is shown in Figures 1 and 2. Figure 1 shows the effect of modality for true sentences. Figure 2 shows the same effect for false sentences.

Based on established effects from the literature, together with a visual inspection of the ERP waveforms, we divided the analysis into the following time windows after critical word onset: 190–300 ms to capture the N1–P2 complex, 325–400 ms to capture a smaller peaked N400 effect, 300–500 ms to encompass the N400 window, and 600–850 ms for late effects. The results for each time window are discussed in turn below. Figures 1 and 2 illustrate the effect of modality switching for each of these four time windows.

For each time window, a fully within participants three-way analysis of Modality switch (match, mismatch), Veracity (true, false), and Region (anterior, posterior) was conducted. This was followed by planned comparisons of (i) Modality switch for true sentences, (ii) Modality switch for false sentences, (iii) Veracity for matched sentences, and (iv) Veracity for mismatched sentences.

### First time window: 190–300 ms

This time window was selected to examine the N1–P2 complex. In the overall 2 × 2 × 2 analysis, a main effect of Modality switch was found [*F*(1, 10) = 5.04, MSE = 0.27, *p* < 0.05], where different modality sentences evoked greater positivity than same modality (0.292 vs. 0.242 μV, difference 0.05). A Modality switch by Region interaction [*F*(1, 10) = 5.19, MSE = 7.79, *p* < 0.05] was also found, as well as a Modality switch by Region by Veracity interaction [*F*(1, 10) = 6.35, MSE = 8.79, *p* < 0.05].

**Figure 3:**
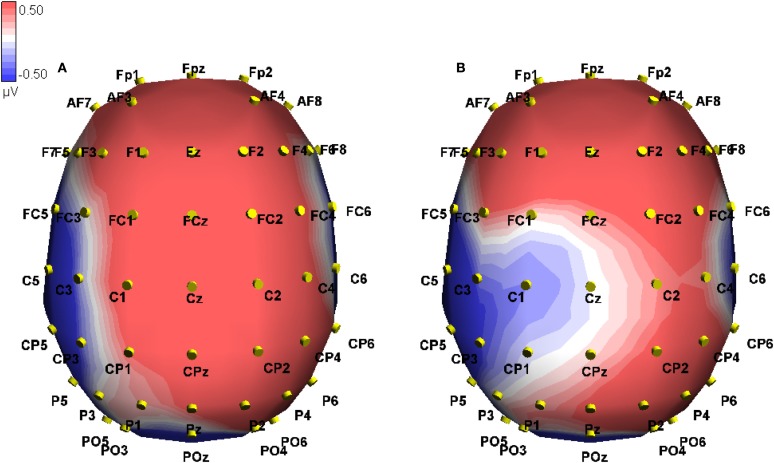
**Event-related potential in microvolts across the scalp at 238 ms post onset of the critical word, approximately at the peak of the difference**. Blue hues indicate negative potentials, red hues positive potentials. The two conditions shown are True-Mismatch **(A)** and True-Match **(B)**.

We investigated both interactions using a simple main effects analysis. For true sentences alone, a Modality switch effect was found across frontal electrodes [*F*(1, 10) = 29.79, MSE = 2.00, *p* < 0.001], where a greater positivity was seen for modality mismatch than match (0.837 vs. 0.180 μV, difference 0.657). Staying with the true sentences, the Modality switch effect was reversed for the posterior electrodes [*F*(1, 10) = 25.33, MSE = 1.48, *p* < 0.01; −0.244 vs. 0.277 μV, difference −0.521; see Figure 3]. For false sentences, no effect of modality switch was found.

Similarly, for modality matched sentences, no effect of Veracity was found. For modality-mismatched sentences, however, a marginal effect of Veracity was found for the frontal electrodes [*F*(1, 10) = 4.23, MSE = 6.75, *p* = 0.067], where true sentences evoked a greater positivity than false (0.837 vs. 0.382 μV, difference −0.455). This effect was reversed in the posterior region (−0.244 vs. 0.192 μV, difference 0.436) but failed to reach significance [*F*(1, 10) = 3.52, MSE = 7.42, *p* = 0.090].

### Second time window: 325–400 ms

This window was selected to examine early but brief N400-like effects. The overall analysis for this window showed only interactions of Veracity by Region [*F*(1, 10) = 7.91, MSE = 17.61, *p* < 0.05] and Veracity by Region by Modality switch [*F*(1, 10) = 9.31, MSE = 19.20, *p* < 0.05].

**Figure 4:**
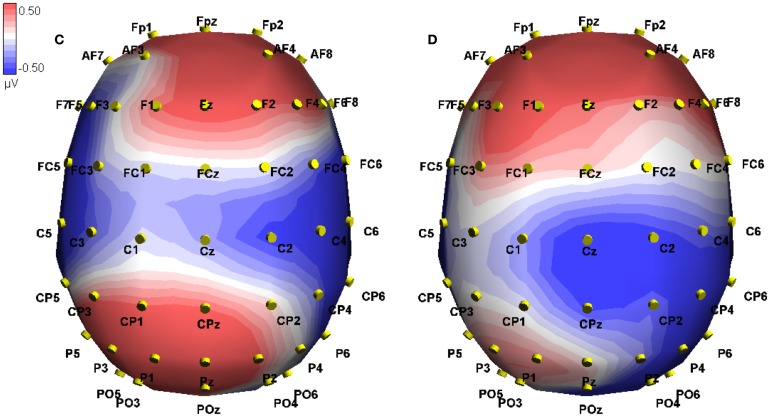
**Event-related potential in microvolts across the scalp at 377 ms post onset of the critical word, approximately at the peak of the difference**. Blue hues indicate negative potentials, red hues positive potentials. The two conditions shown are False-Mismatch **(C)** and False-Match **(D)**.

**Figure 5:**
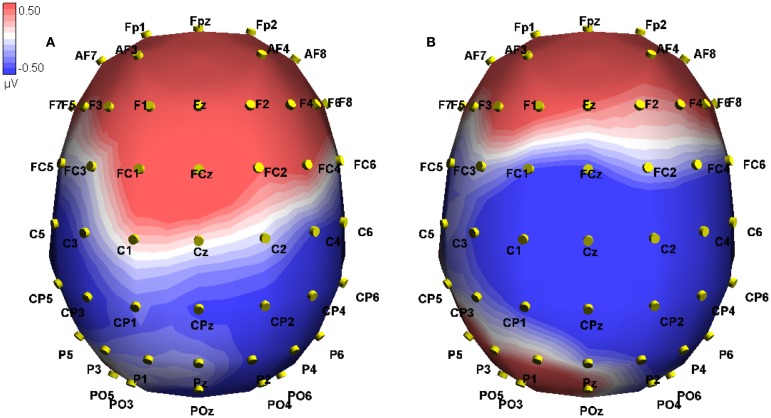
**Event-related potential in microvolts across the scalp at 364 ms post onset of the critical word, approximately at the peak of the difference**. Blue hues indicate negative potentials, red hues positive potentials. The two conditions shown are True-Mismatch **(A)** and True-Match **(B)**.

These interactions were investigated using simple effects. For true sentences, we found a marginally significant effect of Modality switch [*F*(1, 10) = 4.28, MSE = 20.72, *p* = 0.065] in the frontal electrodes, where mismatch showed a greater positivity than match (0.929 vs. 0.126 μV, difference 0.803). This effect was reversed in the posterior electrodes [*F*(1, 10) = 5.30, MSE = 10.30, *p* < 0.05], where greater negativity appeared for the modality-mismatched sentences (−0.590 vs. 0.040 μV, difference −0.63; see Figure 5 below). For false sentences, no effect of Modality switch was found across the frontal electrodes, but was found for the posterior electrodes [*F*(1, 10) = 5.20, MSE = 5.51, *p* < 0.05], where greater positivity was associated with mismatch compared to match (0.465 vs. 0.009 μV, difference 0.456). See Figure 4 for a topographic illustration of this effect.

**Figure 6:**
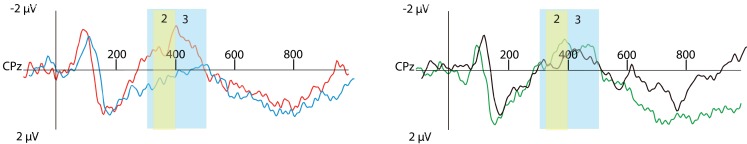
**Event-related potential traces on CPz comparing veracity for matching and mismatching modalities, time locked to onset of the critical word (presented at 0 ms)**. Negative activation is plotted up. The left side shows true (red) and false (blue) sentences in the mismatch condition. The right side shows true (green) and false (black) sentences in the match condition. The limits of the two time windows of interest are indicated (2 = 325–400 ms; 3 = 300–500 ms).

**Figure 7:**
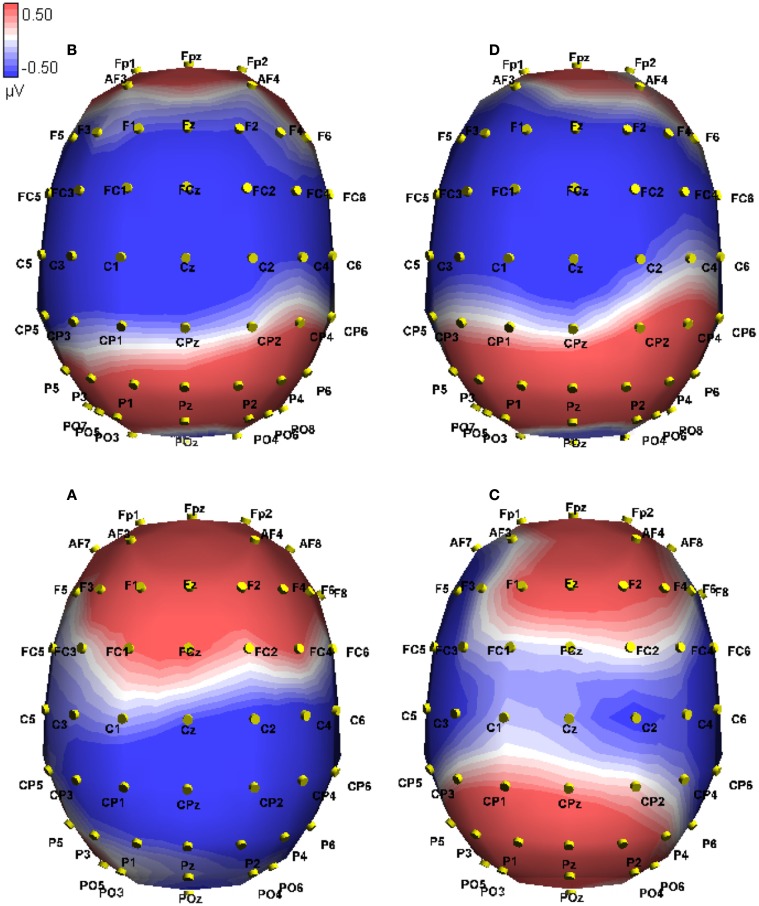
**Event-related potential in microvolts across the scalp at 397 ms post onset of the critical word, approximately at the peak of the difference in the mismatch conditions**. Blue hues indicate negative potentials, red hues positive potentials. The conditions shown are True-Match **(B)**, False-Match **(D)**, True-Mismatch **(A)**, and False-Mismatch **(C)**.

We investigated Veracity for matched sentences and found no effect in either the frontal or posterior regions. However, for mismatched sentences, we found a frontal effect for Veracity [*F*(1, 10) = 24.71, MSE = 6.62, *p* < 0.01], where false sentences elicited a greater negativity than true (−0.162 vs. 0.929 μV, difference −1.091), as well as a reversed posterior effect [*F*(1, 10) = 36.00, MSE = 4.26, *p* < 0.001], where greater negativity was associated with true sentences vs. false (0.465 vs. −0.590 μV, difference 1.055). This is illustrated in Figures 6 and 7 below.

### Third time window: 300–500 ms

We chose this time window to examine N400-like effects. The overall analysis found a main effect of Modality switch [*F*(1, 10) = 5.59, MSE = 0.37, *p* < 0.05], where overall matched sentences showed greater negativity than mismatched (0.100 vs. 0.161 μV, difference 0.061), as well as a Veracity by Region interaction [*F*(1, 10) = 7.78, MSE = 24.12, *p* < 0.05] and a Veracity by Region by Modality switch interaction [*F*(1, 10) = 8.54, MSE = 16.41, *p* < 0.05].

Using simple main effects, we examined Modality switch in the frontal region for true sentences, finding a main effect [*F*(1, 10) = 6.67, MSE = 12.64, *p* < 0.05], where mismatch showed the greater positivity (0.857 vs. 0.074 μV, difference 0.783; see Figure 5. This effect was reversed for the posterior region [*F*(1, 10) = 8.63, MSE = 6.35, *p* < 0.05], where greater negativity was associated with mismatched sentences (−0.509 vs. 0.122 μV, difference −0.631). For false sentences, there was no effect of Modality switch in either the frontal or posterior regions.

There was no effect of Veracity for modality matched sentences in the frontal or posterior regions. However, for mismatched sentences, we found a marked positivity for true sentences in the frontal region [*F*(1, 10) = 25.24, MSE = 6.77, *p* < 0.05; 0.857 μV vs. −0.258, difference −1.115]. For the posterior region, this effect was reversed [*F*(1, 10) = 30.05, MSE = 5.18, *p* < 0.001; 0.555 μV vs. −0.509, difference 1.064]. Figure 6 illustrates this veracity effect at site CPz and Figure 7 illustrates a topographical plot of the veracity effect.

### Fourth time window: 600–850 ms

**Figure 8:**
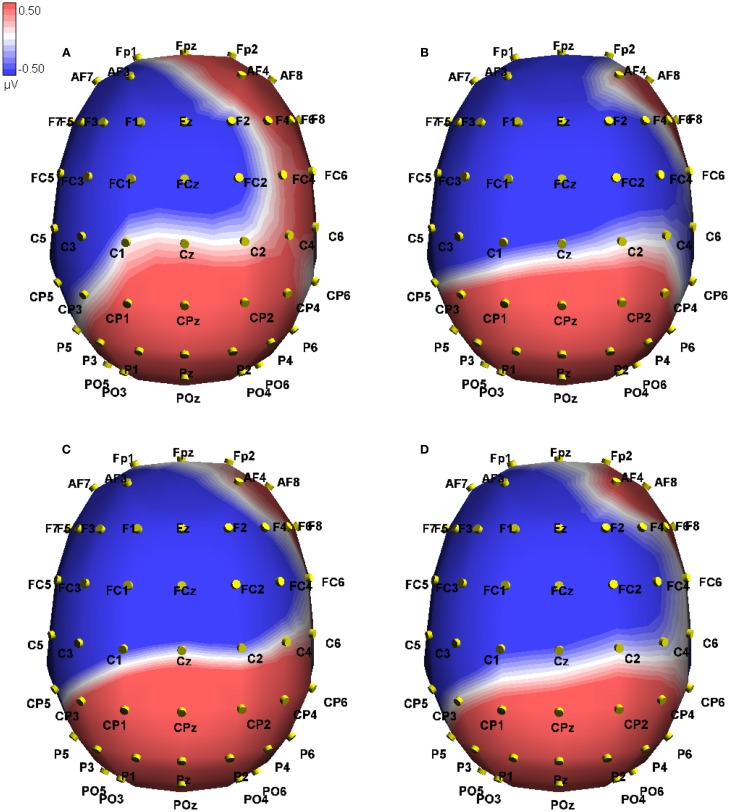
**Event-related potential in microvolts across the scalp at 661 ms post onset of the critical word, approximately at the peak of the difference in the mismatch conditions**. Blue hues indicate negative potentials, red hues positive potentials. The conditions shown are True-Mismatch **(A)** True-Match **(B)**, False-Mismatch **(C)**, and False-Match **(D)**.

This time window was chosen to examine late positive effects. In the overall analysis, we found interactions of Veracity by Modality switch [*F*(1, 10) = 3.71, MSE = 0.64, *p* < 0.05], and Veracity by Modality switch by Region [*F*(1, 10) = 7.06, MSE = 30.12, *p* < 0.05]. See Figures 1 and 2 for a plot of representative electrodes within this time window and Figure 8 for topographical plot of this effect.

Simple main effects were used to examine the effect of Modality switch in the frontal region for true sentences. We found a marginally significant effect of Modality switch [*F*(1, 10) = 4.59, MSE = 20.98, *p* = 0.058], where matched sentences showed a greater negativity than mismatched (−0.883 vs. −0.046 μV, difference 0.837). In the posterior region, the direction of this relationship was reversed but did not reach significance (*p* = 0.08). We repeated these analyses for false sentences and found a significant effect of Modality switch in the frontal region [*F*(1, 10) = 5.27, MSE = 8.50, *p* < 0.05], where a greater negativity was seen for mismatched sentences (−0.752 vs. −0.181 μV, difference −0.571). For the posterior region, this effect was reversed but did not reach significance (*p* = 0.072).

We next examined the effect of Veracity in the frontal region for modality matched sentences but found no reliable differences. Similarly, we found no reliable veracity differences in modality-mismatched sentences for either region.

### Reaction time data

Participants made a true/false judgment after each sentence was presented. We include an analysis of the reaction time data here for completeness. However, since the reaction time data of Pecher et al. (2003) included three times the number of participants per condition than we have used here, we do not necessarily expect to have enough power to detect all differences. Additionally, in order to minimize movement artifacts during the critical word, participants were not required to give a speeded response (see Procedure for the ERP Study for details). All data were trimmed using a non-recursive criterion of 2.5 SD from the mean, which resulted in a loss of 2.5% (40/1600) of trials. Means and standard deviations are presented in Table 3. A mixed effects regression model was used to take into account the effects of both participants and items on response times (modeled as random intercept effects, cf., Janssen, 2012). For this analysis, we included all correct responses to target sentences. The fixed effect of Modality showed a trend [*F*(1, 1318) = 2.78, *p* = 0.096] whereby slower response times were seen in the Matched compared to Mismatched condition (739.6 and 716.6 ms; *d* = 0.033). There was no fixed effect of Veracity [*F*(1, 1322) = 2.42, *p* = 0.120] or Modality × Veracity [*F*(1, 1323) = 0.92, *p* = 0.337] interaction. The random intercept effect of Participants was significant (*Z* = 2.09, *p* < 0.05) and there was similar trend for Items (*Z* = 1.65, *p* = 0.098). These random intercepts are to be expected, are of no direct theoretical relevance and hence are not discussed. Accuracy was consistently high across all conditions: True-Match 83.5% accurate; True-Mismatch 85.25% accurate; False-Match 86.25% accurate; and False-Mismatch 85.75% accurate. A mixed ANOVA revealed no main effects of Modality [*F*(1, 9) = 0.12, *p* = 0.74], Veracity [*F*(1, 9) = 0.46, *p* = 0.52], or interaction between Modality and Veracity [*F*(1, 9) = 0.56, *p* = 0.47].

**Table 3:** **Average reaction time (ms) and standard deviation for the true/false judgments on target sentences**.

Modality switch	True	False
Matched	742 (119)	737 (96)
Mismatched	737 (121)	696 (117)

## Discussion

The goal of the current study is to explore the modulation of the modality switch N400 effect. Specifically we hope to understand whether this effect is sensitive to linguistic and semantic markers. To realize this goal, we have added the factors negation and veracity to a typical modality switch paradigm, but in this case while simultaneously recording ERPs. We hoped to shed light on two questions. First, do sentences containing negation show a modality switch effect? Finding such an effect would suggest that sentences containing negation are grounded in perceptual systems. Secondly, previous studies on negation indicate that context can affect the processing of negation. Specifically, when negation is used within a supporting context, processing costs of using negation are minimal. Can matching modality information similarly change the processing of negation? In short, our results indicate that the answer to both of these questions is “yes.” Sentences containing negation do show a modality switch effect similar to that seen with affirmative sentences. Additionally, the effect of veracity suggests that matching modality information can affect the processing of negation. Specifically, we see a different N400 pattern for veracity when modality matches, but a standard N400 pattern to veracity when modality mismatches. The details of these effects are discussed below in turn. Finally, in the Section “Conclusion,” we speculate what the current results may mean in terms of the role of embodied simulation in language comprehension more generally.

### Modality switch effect for true sentences

The modality switching results of the negated true sentences parallel previous results found with affirmative sentences (e.g., Collins et al., 2011; Hald et al., 2011). An effect of switching modalities was found in all four of the time windows. Specifically, as early as 190 ms after the onset of the critical word (Time window 1), true-mismatched modality sentences led to a greater negativity across the posterior electrodes compared to true-matched modality sentences. This greater negativity for the true-mismatched modality sentences continued across the posterior electrodes, resulting in significant differences in the time windows 300–500 ms (as well as 325–400 ms) and again from 600 to 850 ms. Additionally, across the frontal-central electrodes, it was the true-matched modality sentences that showed greater negativity rather than the true-mismatch modality sentences. This same overall ERP pattern was seen with the true-mismatch vs. true-match sentences in the comparable affirmative experiment (Hald et al., 2011). Likewise, as in Hald et al. (2011), no significant effect of modality was seen in the reaction times for true sentences.

This modality switch effect has been previously explained in terms of the idea that our conceptual system is grounded in modality specific or embodied simulations (Pecher et al., 2003; Hald et al., 2011; see below for more details); the current finding extends the role of embodied simulations to the immediate processing involved in negated sentences. This is interesting for at least three reasons. First, although some previous behavioral evidence suggests that negation is represented in an embodied fashion, via a simulation that notes the absence of negated information (e.g., Kaup and Zwaan, 2003), none of the previous studies have addressed the role of perceptual modalities on negation processing as the sentence unfolds online. Our results indicate that the role of embodied simulation on negation processing can be immediate and online, rather than a delayed process.

Secondly, the embodied account of negation and false sentences as described by Barsalou (1999) would predict similar ERP modulations for the modality switching for both false and negative sentences. That prediction is not supported here. If negated sentences showed a similar pattern to false sentences, one might expect that the effect of modality switching on negated true sentences would be very different than what was found for modality switching in affirmative true sentences. We do not find that here. Instead it appears that modality switching effects are quite similar regardless of whether the sentences are affirmative or negative.

Lastly, the current study provides an additional demonstration of an N400-like effect being sensitive to modality switching. Possibly the amplitude of the ERP in this case serves as an indicator of the ease or difficulty of retrieving stored conceptual knowledge related to a word. This modulation may depend on both the stored conceptual representation as well as the previous contextual information (see Kutas et al., 2006). For example, when a visual context is followed by the target sentence “*Rice isn’t*…,” participants are likely to form expectations that may be biased by the visual context which leads to a simulation which is biased to new visual information. When the sentence continues with a “visual” word, the word is immediately integrated in the simulation. However, when a tactile word is displayed the modality of the simulation has to be changed which leads to the modality switch effect and the observed negativity in the ERP. Before discussing alternative explanations of the modality switching effect in true sentences, a short discussion of our modality switching results with false sentences is necessary.

### Modality switch effect for false sentences

In the current study we have seen a small but significant effect of modality switching for false-mismatched modality sentences compared to false-matched modality sentences in the posterior electrodes in the time window 325–400 ms after critical word onset (Time window 2). Interestingly, the effect of modality switching for the false sentences is opposite to that seen here for true sentences. False sentences led to a greater N400-like effect for the match modality condition compared to the mismatch condition. With the true sentences the mismatch led to a greater N400-like effect compared to the match condition. This finding is different than what was previously seen with false affirmative sentences (Hald et al., 2011), where no significant effect of modality switching was found. However, it should be noted that the pattern of results for the false sentences in the Hald et al. (2011) study mirrors that seen here; it is simply that the effect was not sufficiently robust to reach significance.

Why the effect for false sentences is significant in one study but not in the other cannot yet be fully explained and as such is worth exploring further in future research. However, since the significant effect for false sentences is quite small (a 0.46 amplitude difference) as well as occurring only in a short time window (325–400 ms), it seems likely that the modality switch effect may be more difficult to find when using false sentences. Previously, we discussed the possibility that the null effect with false sentences may be due to a simulation of the sentence failing (see Hald et al., 2011). Specifically, we assumed that participants compared the information from the simulation of the false sentence to background knowledge they have, and when the simulation did not match background knowledge, the simulation failed (also, see Barsalou, 1999, for a discussion of simulations failing with false sentences). However, it was felt at the time that this was not an entirely adequate explanation of falsity, since it seems that making the simulation of the false sentence itself would still show some benefit of a modality match. We felt a more reasonable explanation was that when participants tried to simulate “*the cellar is light*” (an affirmative false sentence example from Hald et al., 2011) out of context they were unable to immediately activate the relevant perceptual/action/emotion information due to limited experience with the information in the sentences. Essentially we claimed that such simulations take longer out of context, and the modality switch effect being a small and subtle effect, is not observed in this case. However, in the current study with false sentences, we did observe such a modality switch effect. This may be due to the negation itself changing the type of perceptual information that is included in the sentences that needs to be simulated. With negated sentences, the individual lexical items that make up the sentences are concepts that we have had extensive experience of being paired together. To illustrate this take the “*Rice isn’t white*” example. Rice typically *is* white. Given this, it may be the case that it is this relationship between the two concepts that allows participants to more quickly simulate the false sentence, which leads to the small, but significant effect of modality switching. The difference in the direction of the effect with regard to false sentences (false-match sentences leading to greater negativity compared to false-mismatch sentences as opposed to true-mismatch sentences leading to greater negativity compared to true-match sentences) may simply be an indication of the falseness of the sentence, but at this point further research is needed to better understand why false sentences lead to the opposite effect in the ERPs compared to true sentences.

Overall, the results indicate an effect of modality switching on the ERPs regardless of whether the sentences are true or false, but the specific effect differs depending upon veracity (true sentences leading to a larger N400 for mismatch compared to match pairs; false sentences leading to a larger N400 for match compared to mismatch pairs). Essentially it seems that when the reader is in the visual modality, they can easily predict/expect from “Rice isn’t…” anything that is in the visual modality except “white.” “White” is particularly unexpected in this context and therefore produces a larger N400. ERP results for modality switching with affirmative sentences are somewhat similar to ERP modulations that have been found for pictures and combined sentence-picture stimuli (Barrett and Rugg, 1990; Ganis et al., 1996). Essentially, we again found a very similar effect. This suggests that negative sentences, like affirmative sentences that refer to a highly salient physical aspect of an object induce ERP effects that are comparable to those effects that have been obtained with pictures (see Hald et al., 2011, for a more detailed discussion of the parallel between results obtained with pictures and those obtained with sentences). Overall, interpreting these results within an embodied cognition framework would suggest that our participants generated a mental simulation of the properties of the object (*Rice isn’t black*), which produced activation that is very similar to actually seeing the object. An intriguing direction for future research would be to determine whether the visual sentences show this effect more robustly than the tactile sentences, as might be predicted by this explanation. Furthermore, by examining other modalities as well as possible actions and emotions we may be able to find specific ERP signatures that are related to the particular modality/action or emotion being simulated. Some suggestion that this may indeed occur comes from Collins et al. (2011), where differential ERP effects were seen for modality switching for visual vs. auditory properties.

### Veracity results for modality mismatch

With affirmative sentences a larger N400 is typically seen for false sentences compared to true sentences (e.g., Hagoort et al., 2004). However, for negative sentences without a context, the pattern of results typically reverses (e.g., Fischler et al., 1983). True sentences lead to a larger N400 compared to false sentences. This suggests that as far as the N400 is immediately sensitive to integrating words into the higher-order representation, people appear to be at first only considering something like *rice* + *black* when trying to comprehend single sentences containing negation (as in the “two-step” theories of negation discussed in the introduction). In line with this idea, we found that the true-mismatched negated sentences elicited a larger N400 amplitude than the false-mismatched negated sentences in Time windows 2 and 3. No differences were seen across the other two time windows. Similarly, in the reaction times we saw that overall (collapsing across modality match/mismatch) false sentences were responded to faster than true sentences. No interaction was seen with modality, an issue that will be discussed below.

It has been suggested that for negation to be processed immediately, like affirmative sentences, a context of plausible denial is necessary (Wason, 1965). A context of plausible denial is when one negates something that may have been mistakenly believed (e.g., “The nurse was not a woman”). In the current study, for the modality-mismatched sentences there was no context to aid the processing of negation, and therefore it is not surprising we obtained results compatible with negation not being immediately processed. As outlined above, when the critical word “black” comes in, the modality of the situation needs to change and this causes a delay. By the time of the N400 window, the modality of the simulation may have switched to visual, but given the negation without context, the simulation is essentially based on “rice” and “black” at this stage and therefore a standard N400 for negated sentences is observed.

### Veracity results for modality match

Independent of the explanation of the modality switching effect, our results for matching modality with negated sentences are interesting for another reason. As discussed in the introduction, studies have typically shown that when negated sentences are presented without a discourse context, it is the true sentences that elicit a greater N400 than the false sentences. For our modality matching sentences, this typical finding disappears. Instead we see no difference between the true and false sentences when the modality matches, in any of the four time windows. This is the same pattern as we found with the affirmative sentences (Hald et al., 2011), where no effect of veracity was seen when the modality matched. Why might such a robust effect as veracity disappear when the modalities match? We offer the following tentative hypothesis. In the matched modality case, after a simulation that highlights, for example, visual features (*A giraffe is spotted*), simulating *Rice isn’t white/black* benefits from also being in the visual modality. As discussed in the introduction, it is often assumed that in order to determine truth value of negative sentences, people have to first suppose an “inner proposition,” in this case something like “*Rice is white/black*.” We would suggest that rather than a proposition *per se*, our results suggest that if an early representation like this occurs, then it is more likely to be an embodied simulation than a proposition. Therefore the modality match allows for a richer simulation to arise more quickly, potentially making both true and false sentences equally easy to process. We propose that this is likely the reason why no N400 difference is seen for the true negated condition compared to the false negated condition, similar to what was seen for affirmative sentences. In combination with the results on affirmative sentences, we propose that matching modality allows for a quicker and broader simulation of relevant properties of the sentence, including support for less likely properties of the sentence. Hence making even a typically false property of an object easier to process (see Hald et al., 2011 for a more detailed explanation of how this works with affirmative sentences).

However, facilitating a simulation in itself does not remove the difficulty of processing negation. If that were the case, the false negated sentences should have elicited a larger amplitude N400 than the true negated sentences. Instead we see no difference between the true and false negated sentences. This is also what we found with affirmative sentences (Hald et al., 2011). It seems likely that our results with modality matching facilitates early processes related to veracity judgment and possibly prior to negation being fully taken into account and before the final veracity judgment has been decided. In other words, it seems that the modality match allows for a simulation that is more “accepting” of a wider variety of possible properties of an object, including the less typical ones. However, this is just speculation at this point and clearly needs to be followed up.

The reaction time pattern corresponds to this ERP pattern. The reaction time difference between true and false sentences appears much smaller in the modality matching condition than in the mismatch condition. However, whilst a trend in response times between modality was observed this was not robust and given the lack of any interaction between modality and veracity we can only speculate that with more participants this pattern may more closely match the pattern seen in the ERPs. As noted in the Results section, we were not expecting a significant effect of modality in the reaction times.

Overall, these results suggest that modality matching modulates the veracity-related N400. This may be similar to how discourse context can modulate the effect of veracity (see Hald et al., 2007). How important modality information is for negation processing is still not fully understood, but it may be the case that modality matching information can act as a form of context, like plausible denial, which allows for more immediate processing of negation. Essentially, if readers expect to stay within a modality, that limits what can be said and hence what might be negated.

### Alternative explanations for modality match effects

Although our results fit well with the idea that readers are creating an embodied simulation grounded in the perceptual systems, whereby a mismatch in perceptual information in the sentences leads to a greater processing load, there are alternative explanations for this finding. One alternative explanation of modality switching is based on the organization of the linguistic semantic system. It could be the case that the linguistic semantic system is organized in such a way that is sensitive to modality information, but is still symbolic. This would mean that this effect is not due to activation in modality specific regions in the brain, but instead is due to a type of semantic priming. That is, semantic priming based on modalities rather than semantic association. In the original study by Pecher et al. (2003) the authors attempted to rule out this possibility by conducting a control study where they looked for modality switching type costs with sentences that matched/mismatched in semantic associations (based on Nelson et al., 1999 norms). For example, they looked at sentence pairs like “Sheet can be spotless – Air can be clean.” compared to “Sheet can be spotless – Meal can be cheap.” Here “spotless” and “clean” are highly associated semantically where “spotless” and “cheap” are not. However they found no priming effect or any effect on errors for “clean” compared to “cheap.” There was no cost to switching between sentences that matched or mismatched semantically. This is not too surprising since it has long been known that lexical semantic priming effects are typically very short lived and are not sustained past 1–2 intervening words (e.g., Zwitserlood et al., 2000). Nonetheless, the results of the priming control study further supported the idea that the modality switch costs were due to modality specific information predicted by an embodied model of cognition, rather than priming of symbolic symbols organized by modality (see also Van Dantzig et al., 2008; Oosterwijk et al., 2012; for additional results suggesting that the modality switch effect is not due to semantic priming alone).

However, a recent study by Louwerse and Connell (2011) suggests that instead of relying on an embodied cognition account alone to describe this type of data, they propose that a symbolic and an embodied cognition account can be complementary. They used statistical information about word co-occurrences to predict response times in a modality switch paradigm where participants verified whether properties shared or shifted modalities. Overall, they suggest that two factors contribute to the modality switch effect, semantic priming for modality information (the linguistic word co-occurrence information) and secondarily embodied semantic information. Although our study is not designed to tease apart these differences, what is striking about both the current study as well as the results from Hald et al. (2011) is that the two modalities used (visual and tactile) would not be predicted to show any linguistic priming effects according to Louwerse and Connell (2011). Within their model, they found that the linguistic account did not make such fine-grained distinctions between all of the five modalities. Important for the current paper, visual, and tactile modalities were not distinguished within their linguistic model. Accordingly, this would mean that any modality switch effects found here or in Hald et al. (2011) cannot be due to priming of symbols organized by modality, at least if there is no distinction made between visual and haptic modalities. The model proposed by Louwerse and Connell (2011) does not exhaust the possibilities of statistical effects. It is well possible that there are statistical effects they have not picked up that could still be influencing our results. This same basic argument, that linguistic word co-occurrence factors alone cannot account for modality switching effects, was also offered by Connell and Lynott (2011) to account for modality switching costs seen with novel concepts (e.g., *jingling onion*). At present we can only speculate about the influence of statistical word co-occurrence on our results. We believe that there may well be an influence of statistical word co-occurrence information in tandem with an embodied approach, leading to the current results. However, the Louwerse and Connell (2011) approach to linguistic context may not capture statistical patterns at an appropriate level of granularity (contextual frame). The authors define linguistic context as the frequency of first-order co-occurrences of modality specific words (p. 384), which may be insensitive to patterns at other levels. A more sensitive model of word co-occurrences may demonstrate that both statistical properties as well as an embodied approach contribute to our findings with visual and haptic modalities. The difficult task will be to determine under what circumstances statistical information and embodied information/processing differ. For example, one may imagine a situation where word co-occurrence is very low, just because we do not talk about that property of an object often. Nonetheless, in these circumstances, a modality switch effect is still seen due to the embodied simulation. This may be exactly what occurs with novel combinations (e.g., *jingling onion*; Connell and Lynott, 2011). Here there is no word co-occurrence information to rely upon, but a simulation allows us to easily come up with an interpretation of this combination (e.g., An onion that makes a jingling sound when you move it). Maybe the main purpose of the embodied simulation is to support novel combinations, but this is clearly speculative [see Lynott and Connell, 2010 for a review of models of conceptual combinations, including one that utilizes embodied conceptual combination (ECCo)]. There may be many empirical ways of teasing apart embodied and linguistic co-occurrence accounts, and there is already a growing body of evidence suggesting that information like type of stimuli (Louwerse and Jeuniaux, 2010), the particular cognitive task at hand (Louwerse and Jeuniaux, 2010), and the time of processing (Louwerse and Connell, 2011; Louwerse and Hutchinson, 2012) all appear to influence the interaction between statistical information and embodied information.

At this stage there is no evidence for a symbolic system with the complexity needed to account for our results, but we cannot rule out the possibility that evidence for such a system will be found in the future. Our motivation in beginning this project was to better understand the time course of embodied representations with negative true and false sentences rather than understanding the precise nature of how these seemingly embodied representations come about. Furthermore, we feel that a purely statistical account of the type proposed by Louwerse and Connell (2011) is unlikely to accommodate our results. Secondly, by using sentences that matched on modality but varied on veracity, a simple associative priming explanation would predict the same (or similar) findings for both the true and false sentences when they mismatch on veracity. This is not what we found here or in the affirmative study (Hald et al., 2011). Nonetheless, several follow-up studies are currently being conducted to more satisfactorily address whether an associative priming explanation can better account for the data than an embodied framework.

## Conclusion

Overall, our results fit well with idea that during comprehension we construct embodied simulations that are based on the previous discourse information in order to integrate the incoming information with the current simulation (see Glenberg and Robertson, 1999; Zwaan and Madden, 2005; for detailed accounts of how these simulations arise). Specifically our results suggest that the construction of a simulation in one modality for the context sentence can aid the simulation of the target sentence if it is in the same modality. This indicates that the simulation process, which is central to embodied language processing, can be predictive (in line with Barsalou, 2009), and that a stronger prediction can be made when there is no modality switch. We find that it is important to illustrate that judging veracity and understanding negation (linguistic and semantic markers) both seem to be influenced by embodied simulations during language comprehension. However, this is only half of the story. Leaving the conclusion at that is not satisfying; there are already many studies supporting the general idea that embodied simulations underlie language comprehension. We believe that by adding veracity and negation to the list of factors that seem to be influenced by embodied simulations allows us add something new to the larger puzzle of how embodied simulation supports language processing. Specifically, we propose several parameters regarding how embodied simulations support language comprehension in relationship to veracity and negation.

First, our very early effects of modality switching (beginning as early as 190 ms) suggest that the timing of embodied representations can be very fast. This is important because it suggests that the perceptual systems are involved in more than just a late deep-postlexical aspect of semantic processing. Aside from the timing of the effect, we believe that the modality switch effect related to veracity is due to an automatic, yet context driven simulation that is made by meshing the affordances of (i.e., Gibson, 1979) and world knowledge about the objects and actions included in the sentence (and wider discourse when available). Rather than performing some sort of comparison process between the simulation and the situation at hand (as Barsalou, 1999 proposes), instead we propose that the veracity judgment comes out of the process of building the simulation. When you have a false sentence, a slow down^2^ in the simulation occurs, since the process of meshing the affordances is more difficult due to having less experience with the relevant objects and actions in combination in the real world. In terms of our results, this “slow down” is evidenced by a much smaller modality switch effect. This same slow down occurs when you receive novel compounds (e.g., Connell and Lynott, 2011); that is, they find smaller switching costs with novel compounds. However, one’s ability to consciously determine whether to interpret a slowed simulation as due to falseness or simply due to a new concept that we have little experience with depends on the context. In the context of the current experiment (judging sentences to be true or false), you will reach a “false” judgment from that slowed down simulation. On the other hand, when the context is to come up with a valid interpretation of a novel compound (such as, *jingling onion* in Connell and Lynott, 2011), you will not interpret a “slowed” simulation as an indication of falseness, but instead as a new concept. As discussed, it is possible that the modality match allows for a simulation that is more “accepting” of a wider variety of possible properties of an object, including less typical ones, but this process is more difficult. We do not have the space here to expand on all of the predictions this would make, but for example this would suggest that if we tested novel compounds with ERPs, we should find a similar modulation of the ERP for novel compounds as we see here for false sentences: namely, a much smaller effect of modality switching. Furthermore, this “slower” simulation may be the locus of the opposite amplitude switch effect seen in the false sentences, but further research is needed to confirm whether this is the case or not. Lastly, in relationship to negated sentences, we believe that understanding negation depends on the same simulation process described above for veracity. However, unlike veracity, the correct interpretation of negation needs a different type of contextual support and it does not always fall out of the particular context/task demands in the same way that it may do for judging veracity vs. understanding novel compounds. Instead there may be a need for a second process of negating information that is already simulated (as proposed by Kaup et al., 2007a) when there is not much contextual support. However, when there is supporting discourse context and/or supporting world knowledge or in our case, modality matching, the simulation may be able to immediately negate the relevant information while building the simulation (in line with Nieuwland and Kuperberg, 2008). We believe the lack of veracity effects on our negated modality matched sentences may be an indication of the initial steps in this simulation process that could lead to immediate negation during the simulation.

## Conflict of Interest Statement

The authors declare that the research was conducted in the absence of any commercial or financial relationships that could be construed as a potential conflict of interest.

## Footnotes

^1^We assume that this would also be the case for motor and emotional systems (i.e., a mismatch effect would occur for negated sentences containing action or emotional information). However, as the current study only looks at perceptual modalities, we can only conclude that sentences containing negation are grounded in perceptual systems.

^2^When we use the term “slow-down” or slowed simulation here, what we mean is that reaching a final simulation may be slower, however it may be more accurate to describe this as more difficult. Additional research is needed to better determine the best way to characterize this type of simulation.
